# Pharmacokinetic Properties of Cytokines in Their Targeted Delivery Based on Autologous Erythrocyte Pharmacocytes

**DOI:** 10.5195/cajgh.2014.184

**Published:** 2014-12-12

**Authors:** Zhaxybay Zhumadilov, Kulzhan Berikkhanova, Alexander Gulyayev, Zarina Shulgau, Dilbar Ibrasheva, Zhanybek Bokebaev, Nadiar Mussin, Talgat Nurgozhin

**Affiliations:** Center for Life Sciences, Nazarbayev University, Astana, Kazakhstan

**Keywords:** cytokine delivery, autologous erythrocytes, pharmacocytes

## Abstract

**Introduction:**

Using autologous erythrocytes as drug carriers for targeted delivery of cytokines to the sites of inflammation could potentially provide new opportunities for treatment of patients with purulent diseases. The targeted characteristic of erythrocytes is associated with the nature of purulent inflammation, where a large amount of erythrocytes is phagocytized and drugs encapsulated into the erythrocytes could be easily released. On the other hand, autologous erythrocytes meet all the criteria for the ideal drug carrier. They are nontoxic, not immunogenic, and able to bear a large number of drug molecules while preserving an original conformation of the drugs. Thus, in this study, we aimed to analyze pharmacokinetic profiles of IL-1β encapsulated into erythrocytes’ ghosts (pharmacocytes) in comparison to intravenously injected free IL-1β.

**Material and methods:**

Albino rats were randomly divided into two groups, each group receiving a different kind of IV injection via the tail vein. Group A (control) received 500 μg of free IL-1β, and group B received an injection of 1 ml of pharmacocytes loaded with 500 μg of test substance. At fixed time points after injection (15, 30, 60, 180, 540, 720, and 1,440 minutes) serum samples were collected. Homogenates of liver, spleen, lung, heart, kidney, and adipose tissue were obtained 24 hours after injections. Concentration of the tested substance in the collected organs and blood plasma were measured by ELISA.

**Results:**

We have observed an increased half-life period (T1/2) for encapsulated IL-1β compared to the control. T1/2 for free IL-1β was one hour, while administration of loaded pharmacocytes allowed the half-life period to increase by more than 15 fold (1,043.40 ± 137.92 min) preserving high level of IL-1β activity in the blood samples up to 24 hours. The increased time of IL-1β presence in the body when administered in the form of pharmacocytes could be explained by reduction of elimination constant (Cel) by 1.6 fold, and clearance (CLel) by more than 100 fold. We also observed an increased concentration of IL-1β in liver, spleen, and lung over at least 24 hours. When administered in free form, IL-1β disappeared from these organs within 6 hours.

**Conclusions:**

Pharmacocytes have shown to improve pharmacokinetic profiles of IL-1β by increasing the half-life period of the cytokine, reducing its clearance and elimination as well as increasing the deposition of the drug in liver, spleen and lungs. These data suggest that pharmacocytes be effective drug carriers for targeted delivery of cytokines to the sites of inflammation and have a potential for improving the treatment outcomes of purulent diseases.

